# The Persian version of the revised dyadic adjustment scale (RDAS): a validation study in infertile patients

**DOI:** 10.1186/s40359-020-0375-z

**Published:** 2020-01-29

**Authors:** Saman Maroufizadeh, Reza Omani-Samani, Mostafa Hosseini, Amir Almasi-Hashiani, Mahdi Sepidarkish, Payam Amini

**Affiliations:** 1 School of Nursing and Midwifery, Guilan University of Medical Sciences, Rasht, Iran; 2 Department of Medical Ethics and Law, Reproductive Biomedicine Research Center, Royan Institute for Reproductive Biomedicine, ACECR, Tehran, Iran; 3 Department of Epidemiology and Biostatistics, School of Public Health, Tehran University of Medical Sciences, Tehran, Iran; 4 Department of Epidemiology, School of Health, Arak University of Medical Sciences, Arak, Iran; 5 Department of Biostatistics and Epidemiology, Babol University of Medical Sciences, Babol, Iran; 6 Department of Biostatistics and Epidemiology, School of Public Health, Ahvaz Jundishapur University of Medical Sciences, Ahvaz, Iran

**Keywords:** Revised dyadic adjustment scale, Marital quality, Reliability, Validity, Infertility

## Abstract

**Background:**

Infertility can have a powerful impact on marital quality. The Revised Dyadic Adjustment Scale (RDAS) is a widely used measure of marital quality. This scale has not been validated in infertile patients. Therefore, this study aimed to evaluate the reliability and validity of the RDAS in a sample of infertile patients.

**Methods:**

The sample of this methodological study consisted of 254 infertile patients referring to a referral infertility clinic in Tehran, Iran. A battery of questionnaires was administered to the participants, including a demographic/fertility questionnaire, the RDAS, the Relationship Assessment Scale (RAS), the Kansas Marital Satisfaction Scale (KMSS), the Couples Satisfaction Index- 4 Item (CSI-4), the Hospital Anxiety and Depression Scale (HADS), and the Perceived Stress Scale-4 Item (PSS-4). Internal consistency of the scale was assessed with Cronbach’s alpha, construct validity was investigated using confirmatory factor analysis (CFA), and convergent validity was examined by correlating the RDAS with RAS, KMSS, CSI-4, HADS, and PSS-4 instruments.

**Results:**

The mean total RDAS score was 49.26 ± 9.34, and 100 patients (39.4%) had marital distress based on the cut-off value of < 48. The second-order three-factor model of the RDAS exhibited an excellent fit to the data, as indicated by χ^2^/df = 2.26; CFI = 0.96; GFI = 0.91; NFI = 0.93; IFI = 0.96; RMSEA = 0.071 and SRMR = 0.050. The RDAS and its subscales revealed satisfactory internal consistency that ranged from 0.664 to 0.847. Convergent validity was confirmed by strong correlations between RDAS scores and scores on the RAS, KMSS, and CSI-4. These correlations also tended to be larger than correlations with measures of HADS-anxiety, HADS-depression, and PSS-4. Among demographic/fertility variables, only infertility duration was found to be correlated to the RDAS.

**Conclusion:**

The RDAS is a reliable and valid inventory for measuring marital quality in infertile patients. Further validation studies are needed to generalize the underlying structure of the scale in various populations.

## Introduction

The particular evaluation of one’s relationship is generally referred to as relationship satisfaction, which is a crucial aspect of life satisfaction [1, 2]. Among several components in the context of relationship satisfaction, marital satisfaction is an essential element and the phenomenon of marriage and family system [3]. Distinct characteristics of marriage heavily influence the quality of marriage, population requirements, sort of behavior towards the partner and personality attributes, attachment style, couples’ families, forgiveness and sacrifice, religion, emotional intelligence, personal health, sexual relations, behaviors and communication patterns such as intimacy, gratitude, aggression and hostile, good self-esteem, truthfulness, and teamwork [4–7]. Different surveys have shown that marriage satisfaction is affiliated with physical and mental health, social and personal health, neuroticism, well-being, happiness, psychological problems, and quality of life [6, 8–12].

Infertility is one of the main areas where marriage quality is heavily impacted. Infertility is a couple’s failure to get pregnant even with regular, unprotected intercourse for at least 12 months [13].. It has been reported that the global prevalence of infertility is 8–12% of couples [14]. The assessment of marital satisfaction among the infertile patient population is challenging regarding multiple psychological and mental health issues like depression, stress, anxiousness, sexual problems, low standard of living, and poorer marital adjustment [15–18].. Concerning the well-known effect of infertility on psychological factors and the hypothesis of a mutual association between infertility and mental health problems, the process wherein depression, stress and anxiety influence infertility seems to be a subject of discussion [14].. Previous surveys have shown that infertility impacts mental well-being, marriage relationships, sexual interactions, and life quality [17].

There are many self-reported instruments for evaluating marriage quality, such as Marital Adjustment Test (MAT), Kansas Marital Satisfaction Scale (KMSS), Dyadic Adjustment Scale (DAS) and its Revised Form (RDAS), Couples Satisfaction Index (CSI), Quality Marriage Index (QMI) and Relationship Assessment Scale (RAS) [19]. To the best of our knowledge, there were no disease-specific scales to assess the marital adjustment for couples with infertility. The 32-item DAS was first created by Spanier in 1976 to measure dyadic adjustment which is composed of dyadic consensus, dyadic satisfaction, dyadic cohesion, and affectional expression [20]. The benefit of DAS is the capacity to identify disputes in pair interactions, to use the dimensions individually and to assist find the most excellent therapeutic or preventive action [21]. Furthermore, there were many issues with the accuracy of distressed and non-distressed samples for at least two of the subscales [22]. Busby subsequently launched the updated edition of DAS in 1995, and scientists were prepared to use it with distressed and non-distressed couples [22]. The advantages of 14-item RDAS are the enhancement of psychometric characteristics, its’ shorter form in comparison to the DAS and includes three aspects, including dyadic consensus dyadic satisfaction and dyadic cohesion [22]. Even though the same length scale instruments are applicable as CSI-16, the RDAS is beneficial because of the possibility of investigating couple relationships more broadly [23]. Although the Persian version of RDAS has been validated among general population in Iran, it is important to evaluate this scale among infertile sample regarding their considerable marital conflicts [24].

Despite the relative strength of the RDAS compared to comparable instruments, it is not clear whether the RDAS is still precise to evaluate patients’ marriage performance with infertility knowledge. The main aim of the present research was to examine the reliability and validity of RDAS in a sample of patients with infertility. In addition, a secondary aim was to examine the relationship between demographic/infertility variables and dyadic adjustment among this population.

## Methods

### Participants and study design

In this methodological study, infertile patients referring to infertility treatment clinic of Royan Institute, Tehran, Iran were invited to participate in the research project. We collected data in the evaluation phase of treatment using the convenience sampling method from February to May 2017.

The data were collected through the use of convenience sampling method from February to May 2017. The eligibility criteria for this study were as follows: (1) infertile couples; (2) legal married couples who are admitted to infertility clinic; (3) 18 years or older; (4) ability to read and write in Persian. Infertility is defined as “the failure to establish a clinical pregnancy after 12 months of regular, unprotected sexual intercourse or due to an impairment of a person’s capacity to reproduce either as an individual or with his/her partner.” [25]. The number of participants required for factor analysis was calculated using the rule of thumb suggested by Guilford [26] and Cattell [27]. They urged researchers to obtain samples of 200 (or 250) observations whenever possible. A total of 254 infertile patients agreed to take part and fill out the instruments altogether.

### Measures

#### Demographic and infertility information

Demographic and infertility information of participants including age, sex, educational level, duration of infertility, cause of infertility, failure of previous treatment, and history of abortion were collected.

#### Revised dyadic adjustment scale (RDAS)

The RDAS is a short form of the original Dyadic Adjustment Scale that measures marital quality [22]. This scale is developed by Busby et al. among clinical and nonclinical couples in USA in 1995 [22]. The RDAS consists of 14 items that comprise three subscales: Consensus (item 1–6), Satisfaction (item 7–10), and Cohesion (item11–14). All items are scored on a 6-point Likert scale ranging from 0 to 5, except for Item 11 which is scored on a 5-point Likert scale ranging from 0 to 4. Total scores can range from 0 to 69, with higher scores indicating more marital quality.

#### Relationship assessment scale (RAS)

The RAS is a brief, 7-item self-administered inventory that measures relationship satisfaction [1].

This scale is developed by Hendrick in USA in 1988. All items are scored on a 5-point Likert scale ranging from 1 to 5, total scores can range from 7 to 35, with higher scores indicating more relationship satisfaction. The Persian version of RAS has been shown to have sound psychometric properties in infertile patients [28].

#### Kansas marital satisfaction scale (KMSS)

The KMSS is an ultra-brief, 3-item, self-administered inventory that measures marital satisfaction [29]. This scale is developed by Schumm et al. among sample of married mothers in 1983 [29]. All items are scored on a 7-point Likert scale ranging from 1 to 7. Total scores can range from 3 to 21, with higher scores indicating more marital satisfaction. The Persian version of KMSS has been shown to have sound psychometric properties in infertile patients [30].

#### The couples satisfaction index- 4 item (CSI-4)

The CSI-4 is an ultra-brief self-administered inventory derived from the original 32 item CSI (CSI-32) that measures relationship satisfaction [31]. This scale is developed by Funk and Rogge among online sample of respondents in USA in 2007 [31]. The first item is scored on a 7-point Likert scale ranging from 0 to 6, and the other three elements are scored on a 6-point Likert scale, ranging from 0 to 5. Total scores can range from 0 to 21, with higher scores indicating more relationship satisfaction.

#### Hospital anxiety and depression scale (HADS)

The HADS is a commonly used self-administered inventory consisting of 14 items designed to measure both anxiety (HADS-A, seven items) and depression (HADS-D, seven items) [32]. This scale is developed by Zigmond and Snaith in 1983 for use in clinical and nonclinical samples [32]. All items are scored on a 4-point Likert scale ranging from 0 to 3. Both subscale scores can range from 0 to 21, with higher scores indicating more anxiety and depression. The Persian version of HADS has been shown to have sound psychometric properties in infertile patients and widely used in this population [16, 33].

#### Perceived stress Scale-4 item (PSS-4)

The PSS-4 is an ultra-brief self-administered inventory derived from the original 14-item PSS (PSS-14) that measures perceived stress [34]. It introduced by Cohen and collogues in 1988 for situations requiring a very short scale or telephone interviews [34, 35]. All items are scored on a 5-point Likert scale, ranging from 0 to 4. Total scores can range from 0 to 16, with higher scores indicating more perceived stress. The Persian version of PSS has been shown to have sound psychometric properties in infertile patients and adults with asthma [36, 37].

#### Statistical analysis

The confirmatory factor analysis (CFA), with the maximum likelihood estimation method, was performed to investigate the factor structure of the RDAS. Overall model fit was assessed using several goodness-of-fit indices including the chi-square/degree of freedom (χ2/df), the comparative fit index (CFI), the goodness of fit index (GFI); the incremental fit index (IFI); the normed fit index (NFI); the root mean square error of approximation (RMSEA), and the standardized root mean square residual (SRMR). Values of χ^2^/df < 3, CFI, GFI, NFI, and IFI > 0.95, and RMSEA and SRMR< 0.08 indicate good fit to the data [38–41]. Cronbach’s alpha was used to examine the internal consistency of the scale. The convergent validity of the scale was assessed by examining the relationship between RDAS scores and scores on the other measures of marital quality, HADS, and PSS-4. Additionally, to explore the relationship between RDAS scores and demographic/fertility variables, the Pearson correlation coefficient, independent t-test, and one-way ANOVA were used.

All data analyses were performed using SPSS for Windows, version 16.0 (SPSS Inc., Chicago, IL, USA), except for the CFA, which was conducted using LISREL 8.80 (Scientific Software International, Inc., Lincolnwood, IL, USA).

## Results

### Participant characteristics

The demographic and fertility characteristics of infertile patients are outlined in Table 1. The average age and infertility duration of the participants were 32.09 (SD = 6.55) and 4.85 (SD = 3.73) years, respectively. Of the patients, 55.5% were female, 36.2% were university-educated, 35.8% had male factor cause of infertility, 49.6% had a failure in previous infertility treatments.

**Table 1:** Demographic and fertility characteristics of the infertile patients (*n* = 254)

	mean ± SD or n (%)
Age (years)	32.09 ± 6.55
Sex	
Male	113 (44.5)
Female	141 (55.5)
Educational level	
Primary	61 (24.0)
Secondary	101 (39.8)
University	92 (36.2)
Duration of infertility (years)	4.85 ± 3.73
Cause of infertility	
Male factor	91 (35.8)
Female factor	55 (21.7)
Both	49 (19.3)
Unexplained	59 (23.2)
Failure of previous treatment	
No (First Treatment)	128 (50.4)
Yes	126 (49.6)
History of abortion	
No	194 (76.4)
Yes	60 (23.6)

SD: Standard deviation

### Descriptive statistics of the RDAS

Descriptive statistics and reliability analysis of the RDAS are given in Table 2. The mean RDAS total score was 49.26 ± 9.34. Based on the cut-off value of < 48, 100 patients (39.4%) had marital distress.

**Table 2:** Descriptive statistics and internal consistency of the RDAS

Item	Mean	SD	Corrected item-total correlation				Alpha if item deleted			
			Consensus	Satisfaction	Cohesion	RDAS	Consensus	Satisfaction	Cohesion	RDAS
1	4.04	1.11	0.292			0.263	0.829			0.850
2	4.20	0.96	0.664			0.575	0.749			0.833
3	3.97	1.03	0.672			0.551	0.744			0.834
4	3.98	1.08	0.542			0.472	0.773			0.838
5	3.94	1.09	0.635			0.606	0.751			0.831
6	3.86	1.19	0.579			0.556	0.765			0.833
7	4.30	1.05		0.681		0.547		0.802		0.834
8	3.44	0.94		0.663		0.584		0.810		0.833
9	4.39	0.99		0.681		0.590		0.802		0.832
10	3.33	0.99		0.695		0.554		0.795		0.834
11	2.17	0.99			0.367	0.368			0.650	0.844
12	2.55	1.33			0.534	0.553			0.538	0.833
13	2.14	1.60			0.562	0.441			0.507	0.844
14	2.94	1.54			0.360	0.379			0.662	0.848
Consensus	23.99	4.59								
Satisfaction	15.46	3.29								
Cohesion	9.81	3.91								
RDAS	49.26	9.34								
Cronbach’s α							0.801	0.844	0.664	0.847

RDAS: Revised Dyadic Adjustment Scale; SD: Standard Deviation

### Correlations between RDAS subscales

Table 3 presents relationships among RDAS subscales. The findings indicate that the correlations between the RDAS subscales are statistically significant. These correlations demonstrate a link between the subscales, but not so strong that they would be taken as a single entity (thus justifying the existence of the subscales).

**Table 3:** Correlations between the RDAS subscales

	Consensus	Satisfaction	Cohesion	RDAS
Consensus	1			
Satisfaction	0.473	1		
Cohesion	0.401	0.452	1	
RDAS	0.825	0.773	0.774	1

All correlations were significant at 0.001 level

### Internal consistency of the RDAS

Cronbach’s alpha coefficients for assessing the internal consistency of the RDAS were as follows: RDAS total (14 items, α = 0.847), Consensus subscale (6 items, α = 0.801), Satisfaction subscale (4 items, α = 0.844), and Cohesion subscale (4 items, α = 0.664). These values remained stable if one item was deleted (see Alpha if item removed values in Table 2). The corrected item-total correlations were between 0.263–0.606.

### Convergent validity

As anticipated, there were strong correlations between RDAS total scores and measures of RAS (r = 0.688), KMSS (r = 0.667), and CSI-4 (r = 0.591). The RDAS total scores were also negatively correlated with measures of HADS-A (r = − 0.457), HADS-D (r = − 0.483), and PSS-4 (r = − 0.487). Similar results were also obtained for RDAS subscales. An inspection of these correlation coefficients (see Table 4) indicates that RDAS scores correlate more highly with measures of marital satisfaction (i.e., RAS, KMSS, and CSI-4) than with measures of anxiety, depression, and perceived stress (i.e., HADS-A, HADS-D, and PSS-4).

**Table 4:** Correlations coefficients between RDAS scores and other measures of marital satisfaction, and measures of anxiety, depression and stress

	RAS	KMSS	CSI-4	HADS-A	HADS-D	PSS-4
RDAS	0.688	0.667	0.591	−0.457	−0.483	−0.487
Consensus	0.521	0.447	0.504	−0.327	−0.361	−0.380
Satisfaction	0.666	0.674	0.543	−0.427	−0.411	−0.492
Cohesion	0.475	0.504	0.365	−0.351	−0.384	−0.305

All correlations were significant at 0.001 level

RDAS: Revised Dyadic Adjustment Scale; RAS: Relationship Assessment Scale; KMSS: Kansas Marital Satisfaction Scale; CSI-4: Couples Satisfaction Index- 4 Item (CSI-4); HADS: Hospital Anxiety and Depression Scale; PSS-4: Perceived Stress Scale-4 Item

### Confirmatory factor analysis

**Fig. 1:**
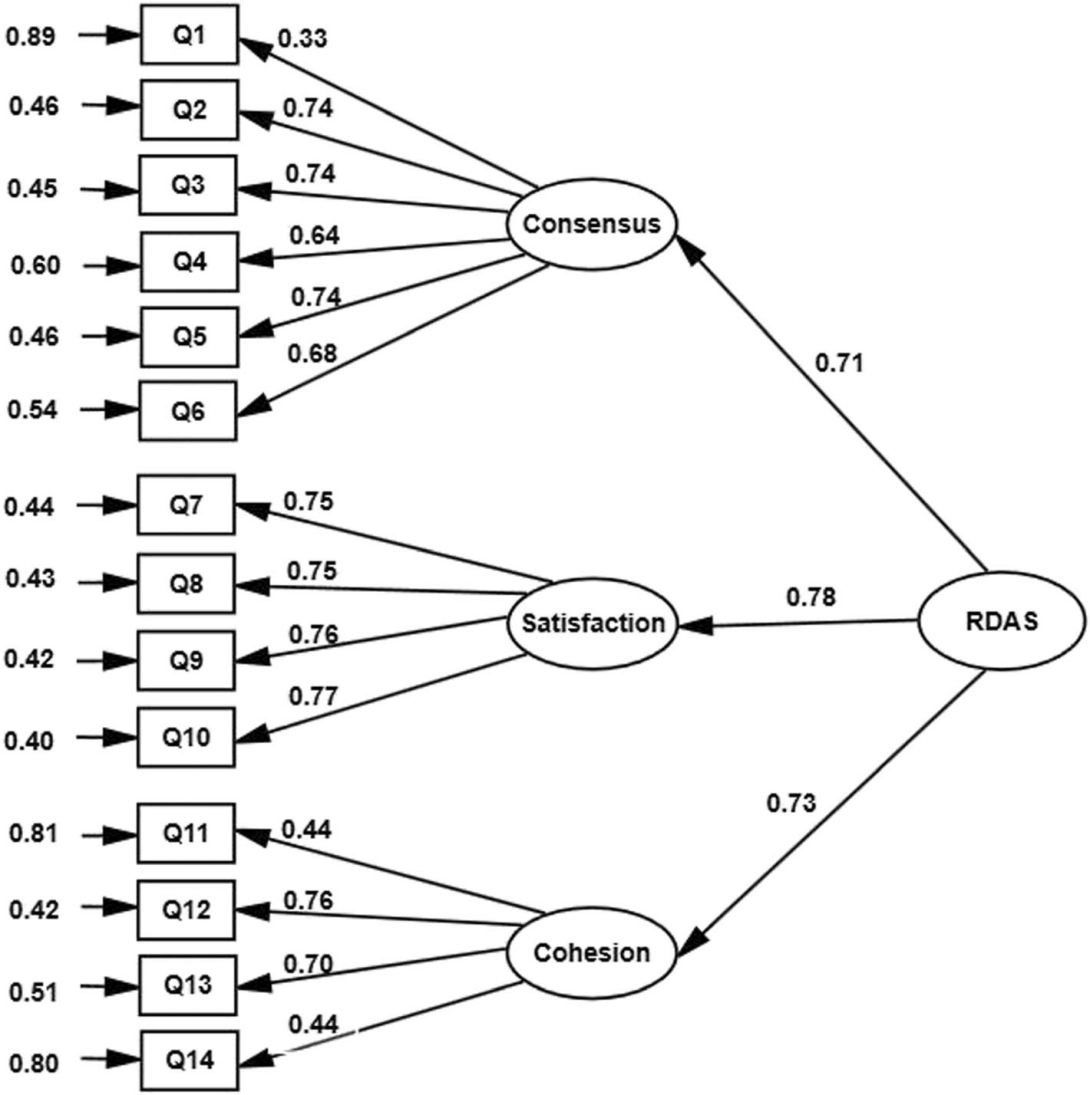
The second-order three factor model of RDAS in a sample of infertile patients

To investigate the factor structure of the RDAS, the CFA was carried out. According to the goodness of fit indices, the fitness of the second-order three-factor model of RDAS was satisfactory (χ^2^/df = 2.26; CFI = 0.96; GFI = 0.91; NFI = 0.93; IFI = 0.96; RMSEA = 0.071 and SRMR = 0.050). As presented in Fig. 1, all standardized factor loadings were significant and in the expected direction, ranging from 0.33 to 0.77.

### Relationship of the RDAS scores with demographic characteristics

As shown in Table 5, a significant but low negative correlation was found between RDAS total scores and duration of infertility (r = − 0.176, *P* = 0.005). Patients who had a failure in previous treatment obtained lower RDAS scores compared to patients undergoing first treatment, but this difference was not statistically significant (*P* = 0.056). Age, sex, educational level, cause of infertility, and history of abortion were not related to RDAS scores.

**Table 5:** Relationship of RDAS total scores with demographic/fertility variables in infertile patients

	mean ± SD or r	P
Age (years)	−0.058	0.354
Duration of infertility (years)	−0.176	0.005
Sex		0.756^†^
Male	49.46 ± 9.88	
Female	49.09 ± 8.92	
Educational level		0.565^‡^
Primary	48.21 ± 9.35	
Secondary	49.83 ± 9.56	
University	49.32 ± 9.14	
Cause of infertility		0.056^‡^
Male factor	47.53 ± 9.99	
Female factor	51.71 ± 8.73	
Both	50.20 ± 9.04	
Unexplained	48.85 ± 8.73	
Failure of previous treatment		0.056^†^
No (First treatment)	50.37 ± 9.22	
Yes	48.13 ± 9.37	
History of abortion		0.260^†^
No	49.62 ± 9.12	
Yes	48.07 ± 10.01	

SD: Standard deviation; r: Correlation Coefficients

^†^Independent t test

^‡^One-way ANOVA

## Discussion

This study examined the psychometric characteristics of the RDAS in a sample of infertile patients in Iran. In this study the prevalence of marital distress was 39.4%, which is higher than what was reported in primary care patients in Nigeria (30.0%) [42], in couples facing breast cancer (27.0%) [43], and in prostate cancer patients and their partner (16%) [44]. A comprehensive approach, including psychosocial interventions and support, is required to improve the marital quality in these patients.

The RDAS and its subscales demonstrated satisfactory internal consistency, and alpha value did not increase after deleting any one of the items. All corrected item-total correlations were also within an acceptable range, indicating good internal consistency. These findings are consistent with what was reported in previous studies [22, 45, 46].

The second-order three-factor model demonstrated good fit indices in this study, which is consistent with the original research by Busby et al. [22] and in a sample of married adults in Romania [45]. Unfortunately, the literature in which the factor structure of the RDAS has been studied is limited.

Convergent validity of the scale was confirmed by strong correlations between RDAS scores and scores on the RAS, KMSS, and CSI-4 instruments. These findings are following the previous research, which found that the RDAS scores were correlated to other measures of marital quality and satisfaction [24, 47, 48]. These correlations also tended to be larger than correlations with measures of anxiety, depression, and stress.

Among demographic and fertility factors, only infertility duration was significantly related to RDAS scores, as infertile patients with long infertility duration had lower marital quality. This result is consistent with previous research [28, 30]. Besides, similar results have been found in other research on measures of quality of life [49], anxiety, and depression [50–53]. Consistent with a study by Turliuc and Muraru [45], there was no significant difference between males and females in RDAS scores.

There are several limitations to the study that should be considered. First, the present study was conducted only in one center and thus may not be generalizable. Second, the cross-sectional nature of the study design limits our ability to make causal inferences between RDAS scores and demographic/fertility information. Third, due to practical reasons, the test-retest reliability of the RDAS was not assessed in this study. Furth, although the cut-off point is available for the English version, further research is required to determine the cut-off point for the Iranian population.

## Conclusions

In sum, the RDAS is a reliable and valid inventory for measuring marital quality in infertile patients. Furthermore, the CFA finding provides additional support for the three-factor structure of the RDAS and use of the subscales as distinct variables. Nevertheless, future studies should examine the psychometric properties of RDAS in diverse populations, particularly its test-retest reliability.

## Data Availability

The datasets used and/or analyzed during the current study are available from the corresponding author on reasonable request.

## Abbreviations

CFA: Confirmatory Factor Analysis
CFI: Comparative Fit Index
CSI-4: Couples Satisfaction Index- 4 Item
GFI: Goodness of Fit Index
HADS: Hospital Anxiety and Depression Scale
IFI: Incremental Fit Index
KMSS: Kansas Marital Satisfaction Scale
NFI: Normed Fit Index
PSS-4: Perceived Stress Scale- 4 Item
RAS: Relationship Assessment Scale
RDAS: Revised Dyadic Adjustment Scale
RMSEA: Root Mean Square Error of Approximation
SD: Standard Deviation
SRMR: Standardized Root Mean Square Residual
